# *In vitro* biocontrol potential of plant extract-based formulation against infection structures of *Phytophthora infestans* along with lower non-target effects

**DOI:** 10.3389/fmicb.2025.1569281

**Published:** 2025-04-14

**Authors:** Valentin Penaud, Abdelrahman Alahmad, Mout De Vrieze, Mathilde Bouteiller, Miléna Eude, Aude Bernardon-Mery, Isabelle Trinsoutrot-Gattin, Karine Laval, Adrien Gauthier

**Affiliations:** ^1^UniLaSalle, AGHYLE, SFR NORVEGE FED, Mont-Saint-Aignan, France; ^2^Biom InnoV, Saint-Malo, France; ^3^Gaïago SAS, Saint-Malo, France; ^4^Department of Biology, University of Fribourg, Fribourg, Switzerland; ^5^Plant Production Systems, Agroscope, Nyon, Switzerland

**Keywords:** biocontrol, *Phytophthora infestans*, plant extract, sporangia, zoospores, fungi, bacteria

## Abstract

Late blight, caused by *Phytophthora infestans,* is among the most destructive diseases affecting tomatoes and potatoes. The use of synthetic fungicides is becoming increasingly restricted due to the banning of several active ingredients for environmental and health reasons. Moreover, the rise of fungicide-resistant strains is compromising their effectiveness. Solutions for sustainable crop protection are thus urgently needed. Biocontrol products based on plant extracts appear to be a promising solution. This study aimed to evaluate *in vitro* inhibitory potential of a plant extract-based biocontrol product on the different stages of *P. infestans* lifecycle, including mycelial development and, formation and germination of infection structures (sporangia and zoospores). Non-target effects were also assessed using four fungi, three of which were isolated from the phyllosphere, and two ubiquitous bacteria. For this purpose, the formulated product (FV) and the plant extract at different concentrations (PE and CPE) were tested through bioassays. The results show that the mycelial growth of *Phytophthora infestans* was completely inhibited by the FV and less affected by the CPE. Infection structures were more sensitive to PE than mycelia, although FV was consistently the most effective inhibitor. Interestingly, at non-inhibitory doses, zoospore germination exhibited disturbances, such as an increase in abnormal germination phenotypes. Overall, PE showed significant inhibitory potential against the oomycete. FV exhibited a strong impact on mycelium, sporangia, and zoospores at very low concentrations (0.01–0.05%), suggesting an optimized inhibitory effect of PE. Non-target effects of FV on fungal and bacterial growth were observed only at concentrations substantially higher than those required to inhibit *P. infestans in vitro*. This study highlights the strong efficacy of the plant extract-based biocontrol product against the target oomycete, with minimal impact on non-target microorganisms. These findings support its potential as a promising anti-*Phytophthora* agent within integrated late blight management strategies.

## 1 Introduction

*Phytophthora infestans* (Mont.) de Bary, the oomycete responsible for late blight, is historically infamous for triggering the Irish potato famine of 1845. This pathogen continues to inflict significant economic losses on potato and tomato production worldwide, contributing annually to substantial yield reductions (Nowicki et al., 2012). The aggressiveness of *P. infestans* is partly attributed to its rapid asexual reproductive cycle, which relies on the production of two types of spores. At higher temperatures, airborne sporangia can directly germinate on the leaf surface, or they can, at lower temperatures, produce and release motile zoospores. Both processes lead to host tissue penetration, mycelial development, and sporangiophore production, enabling further infection cycles (Fry et al., 2015). Thus, due to these dual infection mechanisms in *P. infestans*, its effective control has become challenging.

The common method for controlling *P. infestans* in potato and tomato crops is synthetic fungicide application. However, repeated use of these chemicals is expensive and has led to the emergence of resistant strains (Fry, 2008). In recent years, fluazinam and mandipropamid resistant strains have emerged in Europe (Schepers et al., 2018; Abuley et al., 2023). Moreover, the non-target effects of fungicides pose a significant risk of altering the structure of microbial communities (Yang et al., 2011; Dela Cruz et al., 2022). To reduce their risks to environmental and human health, the European Commission has increasingly restricted the use of authorized active substances (Haverkort et al., 2008; Marchand, 2023). As a result, environmentally sustainable protection methods are urgently needed to provide effective alternatives for farmers. Among these, biocontrol strategies are increasingly recognized as viable solutions to reduce fungicide usage (Van Lenteren et al., 2018).

Biocontrol products, including microorganisms and natural substances, have shown significant promise for combating late blight and for being incorporated into pest management programs (Hashemi et al., 2022; Wang and Long, 2023). Potential candidates for biocontrol products include either the use of fungi and bacteria or their active ingredients or metabolites, mainly belonging to the genera *Trichoderma* (Yao et al., 2016; Alfiky et al., 2024), and, *Bacillus* and *Pseudomonas*, respectively (Hunziker et al., 2015; Caulier et al., 2018; De Vrieze et al., 2018, 2019; Wang et al., 2020a; Wang et al., 2020b). Plants represent an abundant source of biocontrol products due to their rich antimicrobial compounds, including terpenes, phenolic compounds, phytoalexins, alkaloids and flavonoids. These molecules can affect various microbial structures such as the cell wall, plasma membrane, mitochondria, and disturb pathways involved in DNA and protein synthesis (Cenobio-Galindo et al., 2024). Numerous studies have validated the efficacy of various plant extracts *in vitro* and in greenhouse experiments against late blight (Dorn et al., 2007; Forrer et al., 2017; La Torre et al., 2019; Najdabbasi et al., 2020). Their efficacy was evidenced by the direct effects of some plant extracts on mycelial growth, sporangia germination, and zoospore release. However, these studies rarely investigated the impact of plant extracts on zoospore germination, a key process in leaf infection (Fry, 2008). Like synthetic fungicides, biocontrol products may also exhibit non-target effects. For example, Teixeira et al. (2023) reported the inhibitory potential of garlic skin extract against phytopathogenic fungi and oomycetes. Additionally, Adamczyk et al. (2023) showed that spruce resin, containing terpenes, exhibits broad-spectrum inhibition affecting both oomycetes, among which *Phytophthora* species and various fungal species. Moreover, the formulation of biocontrol products is an essential process in optimizing the efficacy of active substances (Janisiewicz and Korsten, 2002; Hernandez-Tenorio et al., 2022). Incorporating co-formulants, such as surfactants, may enhance the effectiveness of plant- and algae-based extracts for controlling phytopathogens, both *in vitro* and *in planta* (Paris et al., 2016; Rashid et al., 2022).

The aim of the present study was to evaluate *in vitro* effects of a plant extract-based biocontrol product on *P. infestans*. Initially, both the plant extract at different concentrations (PE and CPE) and its formulated version (FV) were tested for their direct effects on mycelial growth, sporangial germination, zoospore release and germination. To understand the mode of action of this biocontrol product, experiments were conducted to assess membrane integrity within infection structures following treatment.

Finally, since this product is intended for foliar application, the potential non-target effects of both biocontrol versions were evaluated on microorganism’s representative of the plant-associated microbial communities and isolated from the tomato and maize phyllosphere in the laboratory. These included three fungal isolates from the phyllosphere and one phytopathogenic fungus, with the impact on the mycelial growth assessed. *Fusarium* sp. and *Cladosporium* sp. (Ascomycota) were selected due to their widespread presence in plant canopies and soil, as well as their diverse lifestyles, ranging from pathogenic to commensal and saprophytic species (Bensch et al., 2012; Nikitin et al., 2023). *Mucor* sp., (Mucoromycota) was included as a representative saprophytic fungus commonly found in soil and decaying plant material (Hoffmann et al., 2013; Pawłowska et al., 2019). The plant pathogen *Sclerotinia sclerotiorum*, responsible for white mold on various crops, was chosen for its agronomic importance and known interactions with biocontrol agents (Bolton et al., 2006). Additionally, two bacterial species, *Bacillus subtilis* and *Pseudomonas fluorescens* were included due to their widespread distribution in plant-associated microbiota and their roles in plant health and disease suppression (Haas and Défago, 2005; Earl et al., 2008). This selection ensures a relevant assessment of potential non-target effects on ecologically and functionally diverse microorganisms that are likely to interact with foliar biocontrol treatments.

## 2 Materials and methods

### 2.1 *Phytophthora infestans* and culture media

The *Phytophthora infestans* (oomycete) 36_A2 strain was obtained from inov3PT (Achicourt, France) and maintained in the dark at 15°C on agar plates supplemented with 10% V8 tomato juice and 0.1% (w/v) calcium carbonate. For the different tests, only plates with actively growing mycelium were used. Sporangia were collected by adding approximately 4 mL of sterile distilled water to the mycelial cultures. The mycelium was collected by scraping the plate surface with a sterile rake and transferring into a tube. To detach the sporangia from the mycelium, the suspension was shaken vigorously (but not vortexed) and then filtered through a 70 μm sieve to harvest only the sporangia. The sporangial concentration was determined using a Malassez cell and adjusted to 3 × 10^4^ sporangia.mL^−1^. Zoospores were collected by inducing a cold shock. Briefly, 10 mL of sterile ice-cold distilled water was added to a *P. infestans* culture. To isolate the zoospores, 8 mL was pipetted from the surface of the suspension and transferred to a Falcon tube. The concentration was adjusted at 7 × 10^4^ zoospore.mL^−1^ using a Malassez cell.

### 2.2 Fungi and bacterial strains and culture media

*Sclerotinia sclerotiorum* 1980 UF-70, kindly provided by Dr. Sylvain Raffaele (INRAE-CNRS, France), was cultured on Potato Dextrose Agar (PDA, Condalab) in the dark at 20°C. Three fungal strains from the laboratory strain library were also used in this study: *Fusarium* sp. and *Mucor* sp. (both isolated from the maize phyllosphere), and *Cladosporium* sp. (isolated from tomatoes phyllosphere). The *Fusarium* strain was grown on PDA, while *Mucor* and *Cladosporium* strains were cultivated on Sabouraud Dextrose Agar (SDA, Condalab). All fungal cultures were maintained at 20°C in the dark. *Pseudomonas fluorescens* CIP 104605 and *Bacillus subtilis* CIP 52.65 T, obtained from the Pasteur Institute (Paris, France) were cultured at 28°C in the dark on Luria-Bertani (LB) agar (1.5%) and Tryptic Soy Agar (TSA), respectively.

### 2.3 Biocontrol product

Two versions of the biocontrol product were tested: *i*. Plant extract at 2.50% (v/v) and at 10% (v/v), referred to as plant extract (PE) and concentrated plant extract (CPE), respectively; and *ii.* A formulated version (FV) consisting of a 2.50% plant extract mixed with co-formulants. The co-formulants included a preservative to maintain the integrity of the plant extract, a sticker to ensure adherence to plant surfaces, a surfactant to enhance application quality and penetration, and trace elements that provide additional nutritional benefits to the plant. This product was provided by Biom InnoV (Saint-Malo, France) and the specific nature of the plant extract and formulation composition are confidential (Patent n° WO 2024/023446 A1).

### 2.4 Effect on microorganism’s growth

The effect of biocontrol products on the growth of oomycetes, fungi, and bacteria was studied using appropriate agar culture media in Petri dishes. Sterilized culture medium, either supplemented with the biocontrol product or untreated (control), was poured into Petri dishes. To evaluate the effect of the biocontrol product on the mycelial growth of the oomycete and the fungi, a plug taken from the edge of an actively growing mycelium culture was placed at the center of the Petri dish containing the biocontrol product (FV, PE, or CPE at different doses) or a control. The tested concentrations ranged from 0.01 to 2.50% (v/v). Petri dishes were incubated in the dark at 20°C for *P. infestans*, *Mucor* sp., *Fusarium* sp., and *Cladosporium* sp., and at 24°C for *S. sclerotiorum*. Pictures were taken at two time points, depending on the growth speed of the mycelium in control plates. The first time point (early) was defined as when the mycelium covered half the Petri dish, and the second (late) was when the mycelium fully covered the entire Petri dish in the control condition. It is important to note that not all tested microorganisms have the same growth rate such as *Cladosporium* sp., (slow growth) and *S. sclerotiorum* (fast growth). The time points were as follows: 7 and 14 days for *P. infestans*, *Fusarium* sp., and *Cladosporium* sp.; 3 and 7 days for *Mucor* sp.; and 2 and 4 days for *S. sclerotiorum*.

The mycelial surface area was measured using ImageJ software and expressed as a percentage of mycelial growth, with the control condition sets at 100% for each time point using the following formula:


$$\begin{array}{l} \mathrm{Percentage of mycelial growth}=\\ \frac{\mathrm{Mycelial growth of each replicat}\;}{\mathrm{Mycelial growth average of controls}}\;\times 100 \end{array}$$

At least two independent experiments were conducted, each with a minimum of five technical replicates.

### 2.5 Effect on bacterial growth

To assess the effect of the biocontrol product on bacterial growth, cultures of *P. fluorescens* and *B. subtilis* were grown overnight on appropriate agar media. The bacterial cultures were then resuspended in sterile physiological water (pH 7.2–7.4) to achieve an optical density (OD) of 0.5 at 600 nm for *B. subtilis* and an OD of 0.8 at 580 nm for *P. fluorescens* (Picot et al., 2001; Massabò et al., 2018). Serial dilutions were performed, and 100 μL of each dilution was spread onto the surface of agar medium, either supplemented with the biocontrol product or untreated (control). Plates were incubated at 28°C in the dark. Colony counts were performed after 24 h for *B. subtilis* and after 48 h for *P. fluorescens*. Only plates with colony counts between 15 and 300 were considered for analysis. The colony-forming unit (CFU) concentration for each plate was calculated using the following formula:


$$CFU/\mathrm{mL}\left(N\right)=\frac{\mathrm{Number of Colonies}}{\mathrm{Volume}\;x\;\mathrm{dilution factor}}$$

The experiment was repeated independently three times, each including three technical replicates.

### 2.6 Effect on infection structures of *Phytophthora infestans*

The effects of the biocontrol product on sporangia and zoospores were studied using 96-well plates. The wells contained 50 μL of sporangia or zoospore suspension, to which 50 μL of diluted FV, PE or CPE were added, resulting in final concentrations ranging from 0.01 to 1.00% (v/v). Control wells contained 50 μL of sterile water and 50 μL of sporangia or zoospores suspension.

Sporangia and zoospore germination were assessed as described by De Vrieze et al. (2020), with modifications. For sporangia germination, the plates were incubated in the dark at 20°C for 24 h. Images per well were captured at 10x magnification using an inverted epifluorescence microscope (EVOS M5000, Invitrogen, Thermo Fisher Scientific). On average, 180 sporangia per well were counted and classified as germinated or non-germinated. For zoospore germination, plates were incubated in the dark at 18°C for 4 h. Images per well were captured at 10x magnification, and an average of 210 zoospores per well were counted and classified by phenotype. The experiment was repeated three times, with three technical replicates per experiment.

The effect of the biocontrol product on zoospore release was studied following the procedure described by Alfiky et al. (2024), with modifications. Plates containing the diluted FV and PE were pre-cooled before the addition of 50 μL of sporangia suspension to each well. To induce zoospore release, the plates were incubated at 4°C for 2 h and followed by incubation at room temperature for 45 min. Images were captured at 4x magnification using a Cytation 5 plate reader (Biotek, United States). Approximately 120 sporangia per well were counted, and the proportions of empty and full sporangia were calculated to measure zoospore release. The experiment was performed three times, with three technical replicates per experiment.

Sporangia and zoospore viability were determined as described by Joller et al. (2020), with modifications. After 4 h of incubation for sporangia or 30 min for zoospore at 20°C in the 96-well plates containing the biocontrol product or controls, propidium iodide was added to a final concentration of 20 μg.mL^−1^. Propidium iodide is a red fluorescent dye that binds to DNA only when membrane integrity is compromised. Images per well were captured using an inverted epifluorescence microscope (λex = 542/20 nm, λem = 593/40 nm, EVOS M5000, Invitrogen, Thermo Fisher Scientific). On average, 140 sporangia and 190 zoospores per well (stained or not) were counted. The experiment was repeated three times for sporangia (with three technical replicates) and four times for zoospores (with two technical replicates). The brightness and contrast of the fluorescent channel photos were adjusted to the same level in order to better visualize the stained sporangia and zoospores. Image analysis of all infection structures was performed using ImageJ software.

### 2.7 Data analysis

After checking data normality and homoscedasticity, a Kruskal-Wallis test was performed, followed by a Wilcoxon-Mann–Whitney multiple comparison test between treatments with a Bonferroni correction. Statistical significance was accepted when the *p* ≤ 0.05. All statistical analyses were conducted using R studio software (2024.04.2).

## 3 Results

### 3.1 Impact on *Phytophthora infestans* mycelial growth

**Figure 1:**
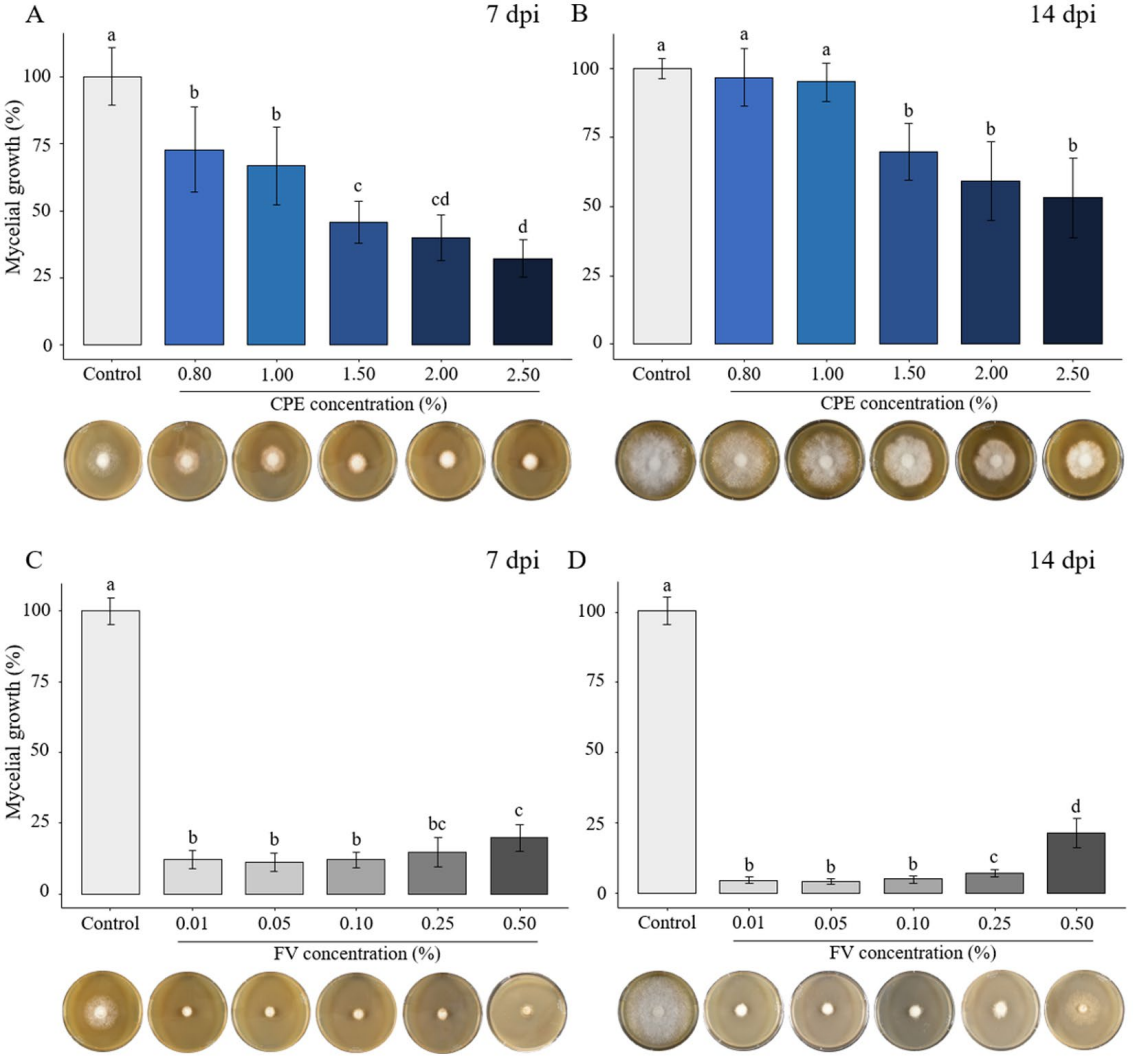
Effect of concentrated plant extract (CPE) and formulated version (FV) at different concentrations on *Phytophthora infestans* mycelial growth at early time (7 days post inoculation - dpi) and late time (14 dpi) (**A,B** and **C,D**, respectively). The percentage of growth was calculated by setting mycelial growth in control at 100%. The bars represent the average percentage growth with standard errors from 12 replicates (pooled from 3 independent experiments, each with four replicates). Representative pictures by time and dose are displayed below each graph. Letters indicate significant differences between treatments (Kruskal-Wallis test, and Wilcoxon rank-sum test for multiple comparisons, *p* ≤ 0.05, *n* = 12).

To investigate the impact of plant extract versions (PE and CPE) and the formulated version (FV) as potential biocontrol agents against *P. infestans*, various doses of PE, CPE, and FV were added to the culture media. Mycelial growth was measured 7- and 14-days post-inoculation (dpi). PE exhibited no inhibitory effect at concentrations up to 2.50% (Supplementary Figures S1A–D). CPE, which is four times more concentrated than PE, showed no effect at concentrations ranging from 0.01 to 0.50% (Supplementary Figures S1E,F). However, at concentrations from 0.80 to 2.50% of CPE, a reduction in growth was observed (Figures 1A,B). At 7 dpi a dose-dependent effect on mycelial growth was observed, with a 30% decrease at lower concentrations and a maximum reduction of 67% at 2.50% (Figure 1A). At 14 dpi, mycelial growth was not significantly inhibited at 0.80 and 1.00%. A significant reduction was maintained only at concentrations above 1.50%, with a maximum decrease of 46% observed at 2.50% dose (Figure 1B). For FV, mycelial growth was reduced by 88% at 7 dpi at the three lowest concentrations (0.01 to 0.10%) (Figure 1C). At the highest dose (0.50%), the reduction was slightly less pronounced but still significant compared to the control, with an 80% reduction (Figure 1C). The same pattern of inhibition was observed at 14 dpi, with a clear difference between the highest and the other doses (Figure 1D). Mycelial growth decreased by 95% at 0.01% and 93% at 0.25%, while at 0.50%, the reduction was 79%. Interestingly, *P. infestans* grew slightly at the highest dose, however the mycelium appeared abnormally gray compared to the control (Figure 1D). In summary, high doses of CPE affected *P. infestans* mycelial growth while FV showed a stronger and more consistent effects at low doses.

### 3.2 Phytophthora infestans infection structures impacted by plant extract and formulated version

#### 3.2.1 Effect on sporangia germination

**Figure 2:**
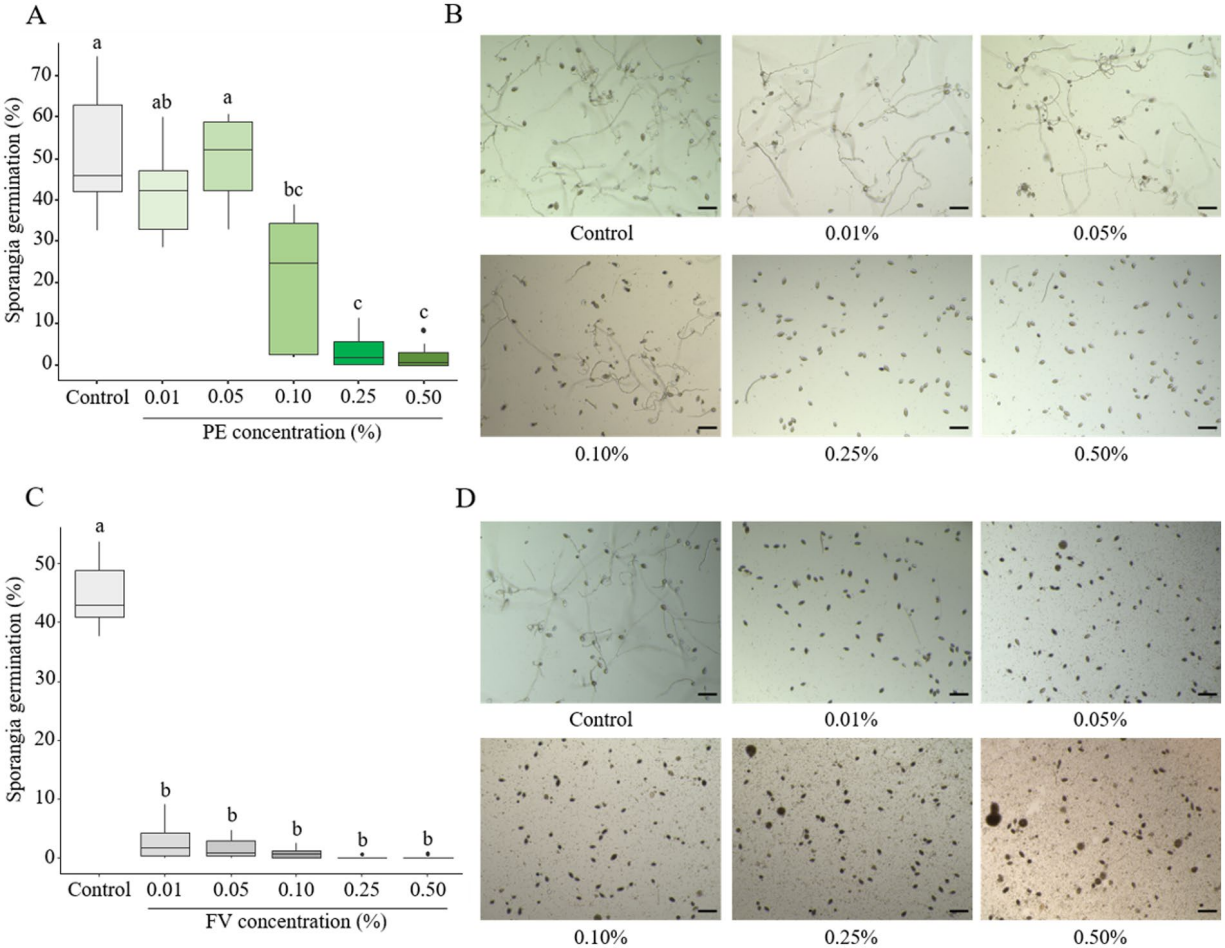
Impact of plant extract (PE) and formulated version (FV) at different concentrations on *Phytophthora infestans* sporangia germination after 24 h of incubation (**A,B** and **C,D**, respectively). Thick lines in boxplots indicate the median of 9 replicates (pooled from three independent experiments, each with three replicates). Letters indicate significant differences between treatments (Kruskal-Wallis test, and Wilcoxon rank-sum test for multiple comparisons, *p* ≤ 0.05, *n* = 9). Representative pictures of *P. infestans* sporangia in the presence of PE (B) or FV (D) at different doses. Pictures were captured using an inverted epifluorescence microscope (EVOS M5000, Invitrogen Thermo Fisher Scientific) at 10x magnification. Scale bars indicate 100 μm.

The sensitivity of sporangia germination was assessed after 24 h of incubation. Low doses of PE (0.01 and 0.05%) showed no impact on sporangia germination (Figures 2A,B). A significant effect was observed starting at 0.10%, though with high variability in response. At the highest concentrations (0.25 and 0.50%), sporangia germination was severely affected, with only 2% being able to germinate (Figures 2A,B). In contrast, FV strongly inhibited sporangia germination at all tested concentrations. At the lowest dose (0.01%), only 2% of sporangia germinated, compared to 40% in the control (Figure 2C). Complete inhibition was observed at 0.25 and 0.50% (Figures 2C,D). In conclusion, sporangia were sensitive to PE, particularly at high concentrations, where sporangia germination was significantly inhibited. Moreover, across all tested doses, FV completely inhibited the germination process.

#### 3.2.2 Effect on zoospore release

**Figure 3:**
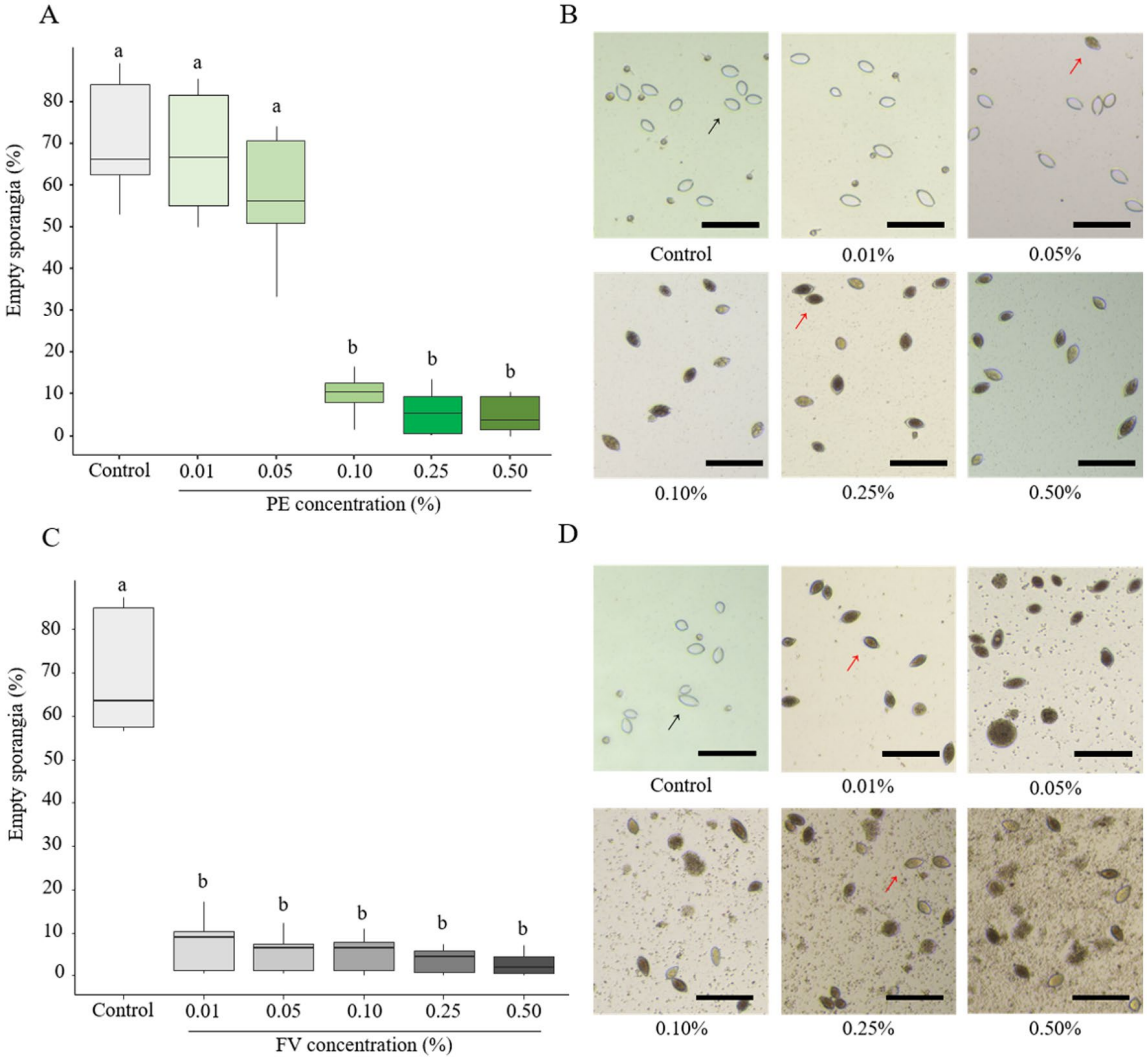
Influence of plant extract (PE) and formulated version (FV) at different concentrations on zoospore release (**A,B** and **C,D**, respectively). Thick lines in boxplots indicate the median of 9 replicates (pooled from three independent experiments, each with three replicates). Letters indicate significant differences between treatments (Kruskal-Wallis test, and Wilcoxon rank-sum test for multiple comparisons, *p* ≤ 0.05, *n* = 9). Representative pictures of sporangia in the presence of PE (B) or FV (D) at different doses. Pictures were captured using a Cytation 5 plate reader (Biotek, United States) at 4x magnification. Black arrows represent empty sporangia and red arrows full sporangia. Scale bars indicate 100 μm.

The impact of PE and FV on zoospore release was evaluated at the same concentrations as in the previous experiments. Empty sporangia (indicated by a black arrow in Figures 3B,D) were identified by the absence of cytoplasmic content and a clearly visible opening at one end. In contrast, full sporangia (indicated by a red arrow, Figures 3B,D) were characterized by retained cytoplasm and a closed sporangium. PE had no impact on zoospore release up to a concentration of 0.05% (Figure 3A). However, at 0.10%, only 9.5% of sporangia were empty. This reduction dropped further to 5% at the highest concentration (0.50%). In contrast, FV exhibited a stronger inhibitory effect. At the lowest dose (0.01%), only 7% of sporangia were empty compared to 68% in the control (Figure 3C). At the highest dose (0.50%), zoospore release was nearly completely inhibited, with only 2% of empty sporangia. In summary, FV affected zoospore release more than PE, with a threshold of 0.01% for FV compared to 0.10% for PE. However, the inhibitory effects of the two versions were comparable at concentrations of 0.25% and higher.

#### 3.2.3 Effect on zoospore germination

**Figure 4:**
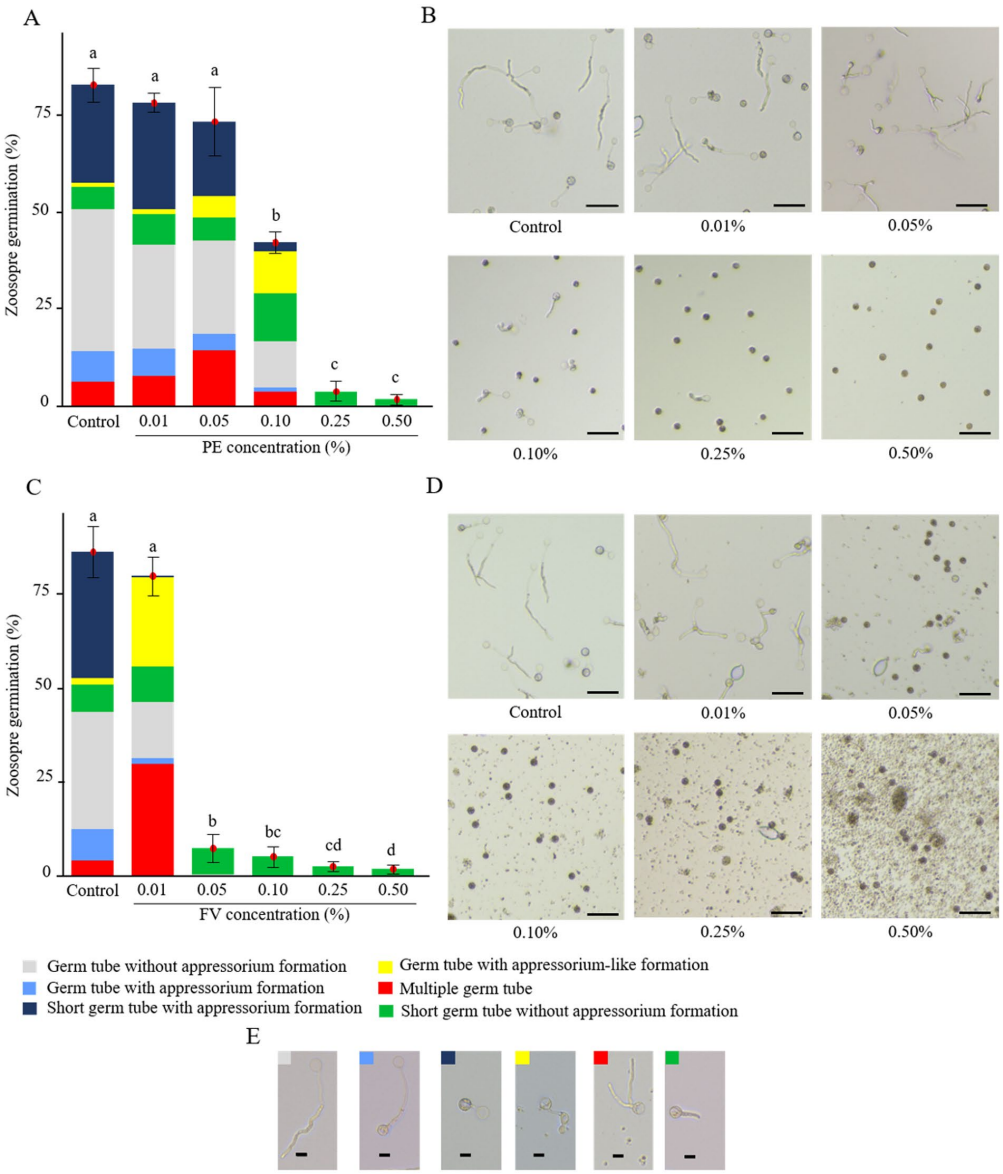
Impact of plant extract (PE) and formulated version (FV) at different concentrations on zoospores germination after 4 h of incubation (**A,B** and **C,D**, respectively). **(A,C)** The bars represent the average percentage of zoospore germination with standard errors of 9 replicates (pooled from three independent experiments, each with three replicates). Letters indicate significant differences between treatments (Kruskal-Wallis test, and Wilcoxon rank-sum test for multiple comparisons, *p* ≤ 0.05, *n* = 9). The colored bars represent the average frequency of the different germination phenotypes observed. **(B,D)** Representative pictures of zoospores in the presence of PE **(B)** or FV **(D)** at different doses and zoospores germination phenotypes **(E)**. Pictures were captured using an inverted epifluorescence microscope (EVOS M5000, Invitrogen Thermo Fisher Scientific) at 10x magnification. Scale bars indicate 50 μm **(B,D)** and 10 μm **(E)**.

The impact of FV and PE on zoospore germination was assessed, revealing significant alterations in both germination rates and phenotypes. Under control conditions, zoospores displayed diverse germination phenotypes, predominantly germination without appressorium formation and germination with short germ tubes with appressorium formation (Figures 4A–E). In contact with PE, total germination rates remained unaffected for the two lowest concentrations (0.01 and 0.05%; Figure 4A). However, at 0.05%, a higher proportion of zoospores with multiple germ tubes and appressorium-like formations were observed (Figures 4A,B). At 0.10%, PE reduced the germination rate by almost half compared to the control. At this concentration, zoospores exhibited the highest proportions of zoospores with short germ tubes without appressorium formation and germ tubes with appressorium-like formation. Moreover, the proportion of zoospores exhibiting short germ tubes with appressorium formation decreased sharply. At higher doses (0.25 and 0.50%), germination was almost completely inhibited, and only short germ tubes without appressoria were observed. In contrast, FV strongly affected the repartition of zoospore germination phenotypes at 0.01%. At this dose, despite the overall germination rate remaining similar to the control, zoospores developed the highest proportions of zoospores with multiple germ tubes and appressorium-like formation (Figures 4C,D). At 0.01%, the predominant germination phenotype observed in the control, characterized by a short germ tube with appressorium formation, was almost absent. At 0.05%, FV significantly reduced zoospore germination, with near-total inhibition observed at 0.50% (Figure 4C). The dominant phenotype at higher FV doses consisted of zoospores with very short germ tubes lacking appressorium formation, similar to the effects observed at the highest doses of PE. Overall, FV had a stronger effect than PE, affecting both germination rates and phenotypes at lower concentrations, while exhibiting similar inhibition outcomes at higher doses.

#### 3.2.4 Effect on sporangia membrane integrity

**Figure 5:**
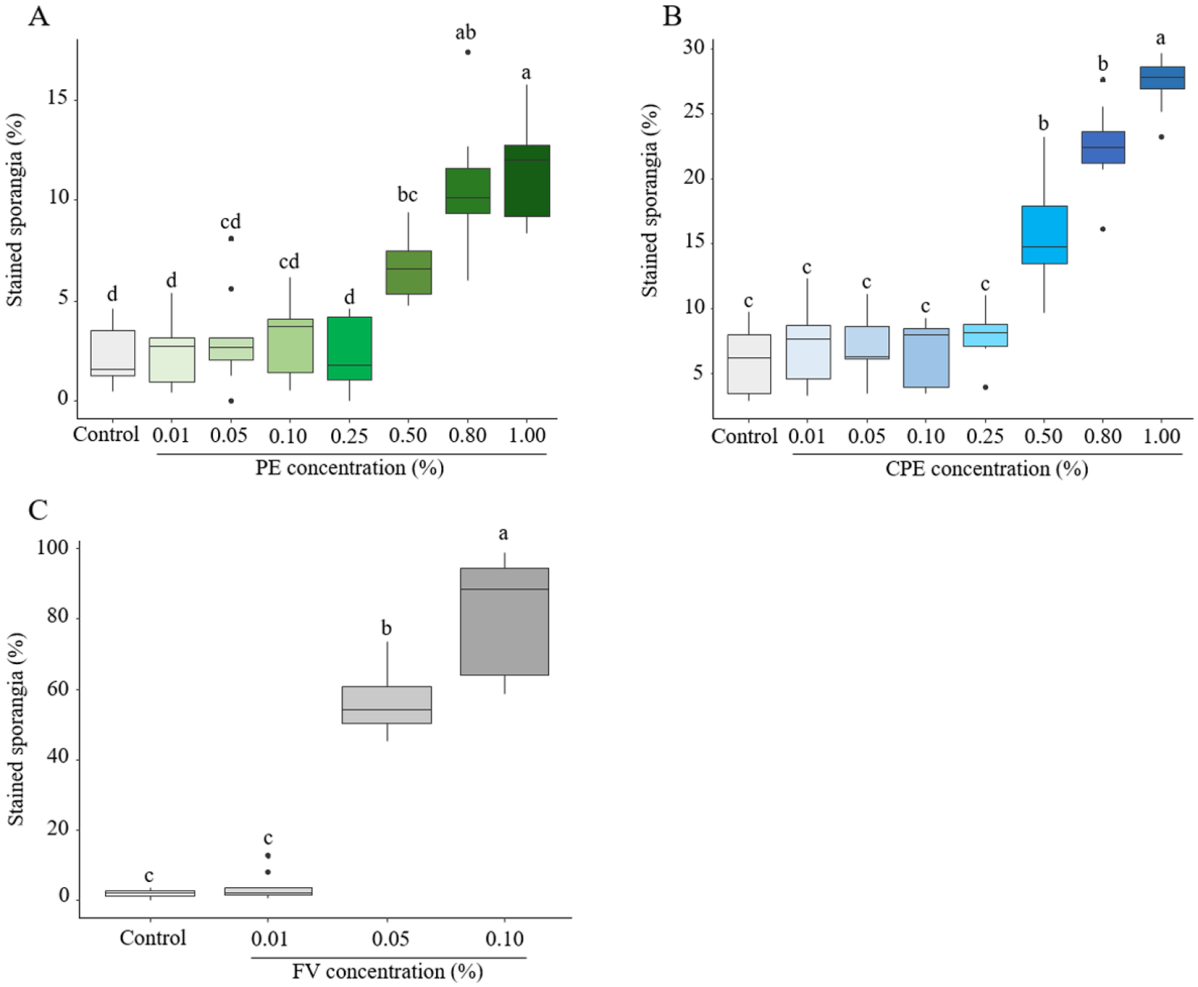
Effect of plant extract (PE), concentrated plant extract (CPE) and formulated version (FV) at different concentrations on the membrane integrity of sporangia after 4 h of incubation, followed by staining with propidium iodide at 20 μg/mL (**A,B** and **C** respectively). Thick lines in boxplots indicate the median of 9 replicates (pooled from three independent experiments, each with three replicates). Letters indicate significant differences between treatments (Kruskal-Wallis test, and Wilcoxon rank-sum test for multiple comparisons, *p* ≤ 0.05*, n* = 9).

To characterize the direct effect of PE, CPE and FV on membrane integrity of *P. infestans*, sporangia were assessed using propidium iodide (PI) staining. PE at concentrations up to 0.25% did not induce observable membrane disruptions in sporangia (Figure 5A; Supplementary Figure S2). However, at 0.50%, PE increased the proportion of PI-labeled sporangia to 6.5%, rising to 11% at 1.00%. A similar trend for CPE at doses ranging from 0.01 to 0.25% was observed (Figure 5B; Supplementary Figure S2), though its impact on sporangial membrane integrity was more pronounced. At 0.50%, around 15% of sporangia were PI-labeled, increasing to 27% at 1.00%. Due to previous results indicating significant effects starting at 0.01% of FV, only the three lowest concentrations of FV were tested. A dose-dependent effect of FV on the proportion of stained sporangia was evident (Figure 5C; Supplementary Figure S2). At 0.01%, no significant impact on sporangia membrane integrity was observed, but at 0.05 and 0.10%, the proportion of stained sporangia increased to over 56 and 80% respectively, suggesting substantial membrane disruption at these concentrations. In conclusion, both PE and CPE exhibited a dose-dependent effect on sporangial membrane integrity at high concentrations (> 0.50%). However, FV had a much stronger impact, significantly compromising membrane integrity at lower doses (< 0.10%).

#### 3.2.5 Effect on zoospore membrane integrity

**Figure 6:**
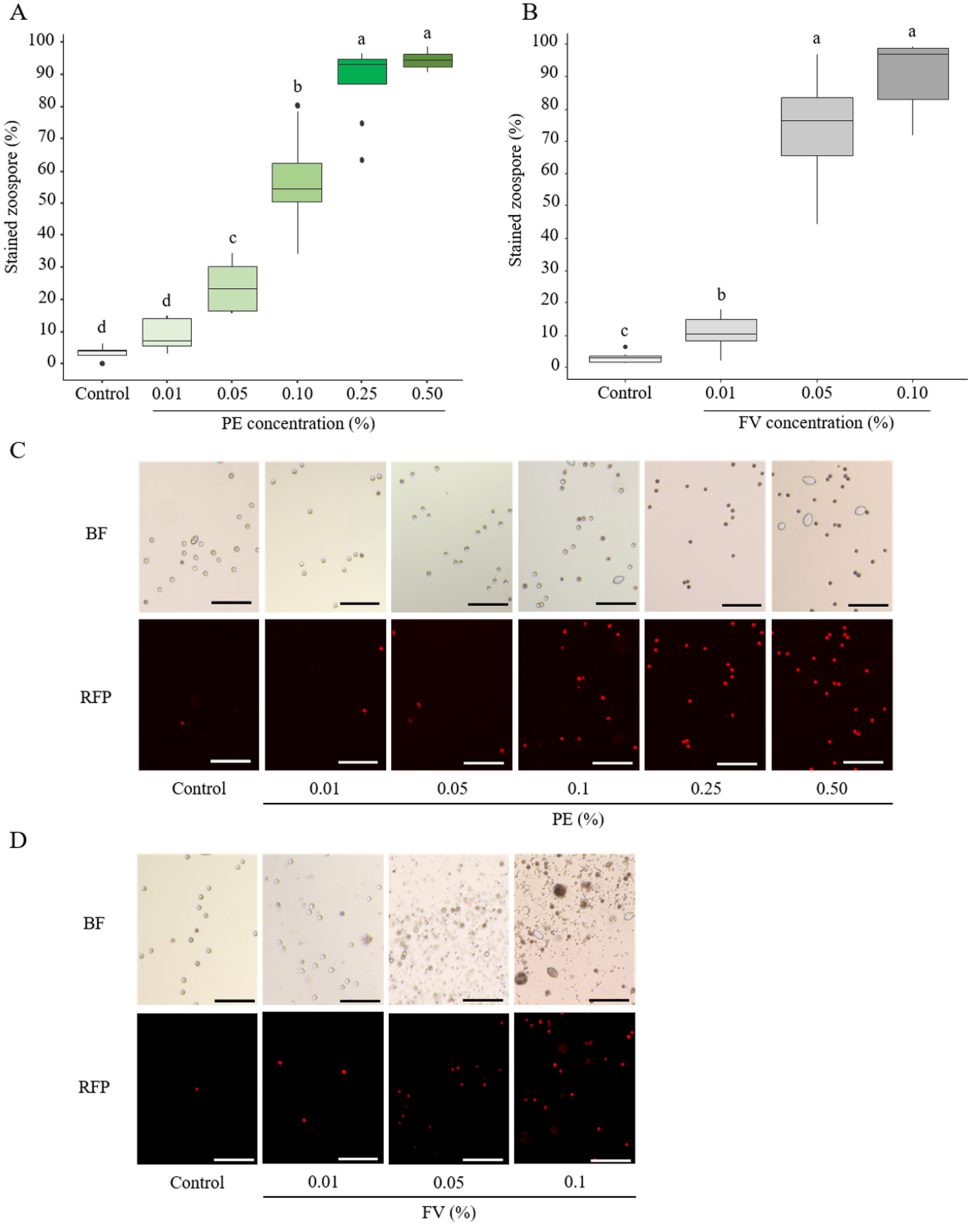
Effect of plant extract (PE) and formulated version (FV) at different concentrations on the membrane integrity of zoospores after 20–30 min of incubation, then stained with propidium iodide at 20 μg/mL (**A,C** and **B,D** respectively). Thick lines in boxplots indicate the median of 8 replicates (pooled from 4 independent experiments, each with 2 replicates). Letters indicate significant differences between treatments (Kruskal-Wallis test, and Wilcoxon rank-sum test for multiple comparisons, *p* ≤ 0.05, *n* = 8). Representative pictures of zoospores in the presence of PE **(C)** or FV **(D)** at different doses. Pictures were captured in bright field (BF) and red fluorescent protein (RFP) channel at 10x magnification using an inverted epifluorescence microscope (EVOS M5000, Invitrogen Thermo Fisher Scientific). Scale bars indicate 100 μm.

In contrast to sporangia, zoospores exhibited marked sensitivity to PE, even at low concentrations (Figure 6A). A clear dose-dependent effect was observed, with intermediate effects appearing at 0.10%, where more than 50% of zoospores were stained. At PE doses above 0.25%, nearly all zoospores exhibited PI staining, indicating severe membrane disruption (Figures 6A,C). Given these findings, further testing with CPE was considered unnecessary. On the other hand, FV induced a slight but significant impact on zoospore membrane integrity at concentrations as low as 0.01% (Figure 6B). At 0.05%, the effect became pronounced, with 74% of zoospores stained. This effect reached a plateau at 0.10%, where nearly all zoospores displayed compromised membrane integrity (Figures 6B,D). In summary, both PE and FV strongly affected zoospore membrane integrity. Zoospores demonstrated a higher sensitivity to FV compared to PE, especially at the lowest tested concentrations.

### 3.3 The formulated version and the plant extract affected fungal mycelial growth differently

To evaluate potential non-target effects of FV and PE, the mycelial growth of several fungal species was examined. Three fungi isolated from the phyllosphere: *Mucor* sp., *Cladosporium* sp., and *Fusarium* sp. were selected to assess possible impacts on microorganisms associated with leaf surfaces. Additionally, *Sclerotinia sclerotiorum*, a fungal pathogen that shares the same host plant as *P. infestans*, was included to provide a comparative analysis with the oomycete. To better assess its potential impact on non-target microorganisms, FV was also tested at an additional higher dose (2.00%). The activity profile of PE on mycelial growth of the tested fungi was distinct. Throughout both observation periods, no significant impact of PE was observed at concentrations up to 0.50% for *S. sclerotiorum* and *Fusarium* sp. (Supplementary Table S1). *Cladosporium* sp. showed a slight growth reduction at 0.50%, but only during the first observation period (Supplementary Table S1). Subsequently, a higher concentration range of PE (0.80 to 2.5%) was tested on these three fungi. After short-term exposure, *Mucor* sp. exhibited sensitivity to PE at 0.25 and 0.50%, with growth reductions of 29 and 53%, respectively, compared to the control (Table 1). A dose-dependent effect was observed in *Cladosporium* sp., *Fusarium* sp., and *S. sclerotiorum*, with initial sensitivity detected at 0.80%, resulting in growth reductions of 28, 18, and 24%, respectively (Table 1). A higher concentration was tested on *Mucor* sp. for comparison with the other fungi. At the high dose of 2.00%, PE caused the most pronounced growth inhibition in *S. sclerotiorum* (80%), while *Fusarium* sp. was the least affected, with a reduction of 42% (Table 1). After long-term exposure, the initial inhibition observed at 0.80% was completely overcome by *Fusarium* sp. and *S. sclerotiorum*, whereas *Cladosporium* sp. remained partially affected, with a sustained 20% growth reduction compared to the control. *Mucor* sp. did not fully recover from the initial inhibition at 0.25%, but its growth was reduced by only 14%. At 2.00%, none of the fungi were able to fully reverse the initial inhibition, although *Fusarium* sp. and *S. sclerotiorum* were able to grow further (Table 1). At 2.50% PE, *S. sclerotiorum* struggled more to overcome inhibition compared to 2.00%, in contrast to *Fusarium* sp., which demonstrated lesser sensitivity at these high doses (Table 1).

**Table 1:** Effect of plant extract and formulated version at different concentrations on the mycelial growth of *Mucor* sp., *Cladosporium* sp., *Fusarium* sp. and *S. sclerotiorum* at early time and late time.

	Plant extract (%)			Formulated version (%)		
	Time point			Time point		
		Early	Late		Early	Late
*Mucor* sp.	Control	100.0 ± 8a	100.0 ± 5a	Control	100.0 ± 8a	100.0 ± 9a
	0.05	90.8 ± 10a	93.9 ± 10ab	0.05	92.0 ± 10a	84.2 ± 14ab
	0.10	92.1 ± 7a	96.9 ± 12ab	0.10	75.0 ± 7b	80.3 ± 8b
0.25	70.9 ± 11b	86.5 ± 7b	0.25	18.0 ± 6c	30.4 ± 14c
0.50	46.8 ± 11c	61.7 ± 9c	0.50	5.0 ± 1d	4.5 ± 0d
2.00	39.4 ± 3c	55.5 ± 2c	2.00	2.0 ± 0e	1.1 ± 0e
*Cladosporium* sp.	Control	100.0 ± 3a	100.0 ± 7a	Control	100.0 ± 6a	100.0 ± 17a
	0.80	72.1 ± 3b	79.9 ± 7b	0.05	97.4 ± 14a	96.1 ± 17a
	1.00	62.6 ± 3c	72.1 ± 8b	0.10	95.2 ± 13a	88.4 ± 14a
1.50	45.9 ± 3d	54.7 ± 6c	0.25	42.8 ± 17b	37.5 ± 11b
2.00	39.2 ± 6d	47.2 ± 5c	0.50	22.9 ± 9b	15.5 ± 9c
2.50	31.5 ± 5e	36.7 ± 5d	2.00	10.8 ± 1c	3.2 ± 1d
*Fusarium* sp.	Control	100.0 ± 2a	100.0 ± 1a	Control	100.0 ± 4a	100.0 ± 3a
	0.80	82.0 ± 6b	99.0 ± 1a	0.05	97.3 ± 4a	92.2 ± 9ab
	1.00	76.9 ± 5b	89.7 ± 8b	0.10	97.0 ± 4a	93.6 ± 7ab
	1.50	68.3 ± 1c	90.1 ± 5b	0.25	84.5 ± 6b	87.7 ± 7b
	2.00	58.5 ± 3d	79.8 ± 6bc	0.50	43.1 ± 5c	70.8 ± 6c
	2.50	51.7 ± 2e	73.9 ± 5c	2.00	11.6 ± 0d	23.3 ± 1d
	*S. sclerotiorum*	Control	100.0 ± 1a	100.0 ± 0a	Control	100.0 ± 3a	100.0 ± 0a
		0.80	75.9 ± 5b	99.7 ± 0a	0.05	94.9 ± 5a	99.6 ± 0a
		1.00	59.4 ± 7c	98.6 ± 3a	0.10	95.1 ± 5 a	99.5 ± 0a
1.50		26.8 ± 1d	86.1 ± 6b	0.25	94.3 ± 5a	99.6 ± 0a
2.00		19.1 ± 1e	60.1 ± 13c	0.50	64.0 ± 5b	99.7 ± 0a
2.50		15 ± 2f	36.9 ± 13d	2.00	2.9 ± 0c	2.5 ± 0b
						

Mycelial growth (%)	≥0; <20	≥20; <40	≥40; <60	≥60; <80	≥80; ≤100

The early time corresponds to 2–7 days post-inoculation (dpi) and the late time to 4–14 dpi. These time points depend on the mycelial growth rate of the fungi (refer to the Materials and methods section). The percentage of growth was calculated by setting mycelial growth in controls at 100%. Values in the tables represent the average percentage of growth with standard errors of the 12 replicates (pooled from three independent experiments, each with 4 replicates). The color scale indicates the percentage of mycelial growth (dark red: high inhibition; white: no inhibition). Letters indicate significant differences between treatments (Kruskal-Wallis test. and Wilcoxon rank-sum test for multiple comparisons, *p* ≤ 0.05, *n* = 12).

After short-term exposure to FV, *Mucor* sp. was again the most sensitive, with its growth partially reduced at 0.10%, unlike the other fungi, where this concentration was ineffective (Table 1). At 0.25%, the growth of *Cladosporium* sp. decreased by 57% compared to the control, similar to *Fusarium* sp., but to a lesser extent (Table 1). *S. sclerotiorum* showed sensitivity starting at 0.50% FV, with a 36% reduction in growth compared to the control (Table 1). At 0.50%, *Mucor* sp. and *Cladosporium* sp. showed the most pronounced growth inhibition, with reductions of 95 and 77%, respectively, compared to the control, while *Fusarium* sp. displayed a more moderate growth reduction of 57% (Table 1). Overall, at 2.00%, the four tested fungi exhibited strong growth reductions (88 to 98%; Table 1). After long-term exposure, the initial growth reduction of *Cladosporium* sp. and *Fusarium* sp. started at 0.25%, although the impact on *Fusarium* sp. was less severe, consistent with earlier observations (Table 1). A similar pattern of sustained inhibition was observed for *Mucor* sp., where growth remained suppressed starting from 0.10%. In contrast, *S. sclerotiorum* completely overcame the initial reduction at 0.50% (Table 1). Notably, at 2.00% FV, *Fusarium* sp. exhibited slight growth progression, accompanied by changes in its appearance and color. Meanwhile, at this concentration, *S. sclerotiorum, Mucor* sp., and *Cladosporium* sp. continued to show strong growth reductions (> 97%; Table 1; Supplementary Figure S3). Overall, FV had stronger antifungal activity than PE, with varying sensitivities observed among the fungal species at high concentrations.

### 3.4 The formulated version and the plant extract exhibited distinct effects on bacterial growth

To further explore the non-target effects, the sensitivity of two ubiquitous bacteria, *Bacillus subtilis* (Gram-positive) and *Pseudomonas fluorescens* (Gram-negative), to PE and FV was evaluated at concentrations of 0.10, 0.50, and 2.00%. After the incubation period, neither bacterial strain showed sensitivity to PE (Table 2). In contrast, a dose-dependent effect was observed with FV. *B. subtilis* showed a 1-log reduction in CFU compared to the control at 0.10%. Notably, *P. fluorescens* showed sensitivity to FV only at 0.50%. At 2.00% FV, no colonies were detected across all dilutions, indicating complete growth inhibition of both bacterial strains. In summary, while PE had no observable effect on either bacterial species, FV demonstrated antibacterial activity at higher doses, with *B. subtilis* displaying greater sensitivity than *P. fluorescens.*

**Table 2:** Effect of PE and FV on bacterial growth of *Bacillus subtilis* and *Pseudomonas fluorescens* 24 h and 48 h of incubation, respectively.

Treatment	Doses (%)	*B. subtilis*	*P. fluorescens*
		CFU.mL^−1^	CFU.mL^−1^
Control	0	1.48 ± 0.274E+08a	6.66 ± 2.18E+08a
PE	0.10	1.43 ± 0.362E+08a	6.23 ± 1.51E+08a
	0.50	1.31 ± 0.286E+08a	7.33 ± 1.98E+08a
	2.00	8.19 ± 4.80E+07a	5.83 ± 0.977E+08a
FV	0.10	1.63 ± 0.375E+07b	6.69 ± 1.59E+08a
	0.50	1.37 ± 0.430E+06c	3.12 ± 0.947E+07b
	2.00	NG	NG

Values represent the average with standard error of 9 replicates (pooled form 3 independent experiments, each with 3 replicates). Letters indicate significant differences between treatments (Kruskal-Wallis test and Wilcoxon rank-sum test for multiple comparisons, *p* ≤ 0.05, *n* = 9). PE: Plant extract, FV: Formulated version, NG: No bacterial growth.

## 4 Discussion

In the context of reducing fungicide usage, exploring sustainable strategies for managing late blight is essential (Popp et al., 2013). Biocontrol agents are emerging as promising alternatives for protecting crops against *Phytophthora infestans* (Hashemi et al., 2022). Beyond understanding their modes of action, assessing their effects on a broad range of organisms is equally important to ensure efficacy and safety. This study applied an *in vitro* approach to evaluate the biocontrol product, focusing on its formulated version (FV), plant extract (PE) and concentrated plant extract (CPE), to investigate their effects on three critical lifecycle structures of this oomycete, and their impact on non-target fungi and bacteria. This dual approach enables us to understand the mechanisms of action while assessing the ecological compatibility of the biocontrol agents, providing insights into their potential role in integrated pest management systems.

### 4.1 The biocontrol product affected mycelial growth of *Phytophthora infestans*

This study revealed strong inhibition of *P. infestans* mycelial growth at low concentrations of FV, while plant extract CPE demonstrated efficacy at higher concentrations (0.80 to 2.50%). The results obtained with CPE suggest that increasing the dose of the plant extract improves its efficacy in a dose-dependent manner. Moreover, these findings highlight the role of the formulation in enhancing the anti-oomycete activity of the plant extract. Among co-formulants, surfactants perform multiple functions, such as enhancing spray quality, improving adhesion, and facilitating the penetration of the active compound into the leaf tissue (Jibrin et al., 2021). Enhanced solubilization of the plant extract in its formulated version may provide a plausible explanation for the observed increase in efficacy. Recent studies have demonstrated that the incorporation of surfactants enhances the efficacy of plant and algal extracts against a range of pathogenic microorganisms. Indeed, Rashid et al. (2022) reported in *in vitro* assays, that formulation was more effective than the crude extract of *Rhus coriaria* in inhibiting the growth of several fungal and bacterial pathogens of tomato, suggesting that surfactants optimize the biocontrol product’s activity. During *in planta* experiments on grapevines Paris et al. (2016) demonstrated a significant enhancement in protection against *Plasmopara viticola* when a surfactant was added to an algal extract. While *P. infestans* exhibited sensitivity to high concentrations of CPE, it showed the ability to overcome the initial inhibitory effects at 0.80 to 1% CPE at 14 dpi. This observation may suggest a potential adaptive response to the plant extract, or alternatively, the decline in inhibitory activity against the oomycete could be attributed to a reduction of the product’s efficacy over time. Similar responses have been observed with prolonged exposure to S-methyl methanethiosulfonate (MMTS) and caraway (*Carum carvi*) essential oil (Quintanilla et al., 2002; Joller et al., 2020). Interestingly, the highest concentration of FV (0.50%) was less effective than lower doses, a difference that became more apparent 14 dpi. This outcome may resemble the paradoxical effect observed in fungi exposed to echinocandins targeting *β*-1,3-glucan synthase, where strains resume growth at higher antifungal concentrations despite being inhibited at lower doses (Bizerra et al., 2011; Juvvadi et al., 2015). This effect is believed to involve an increase in cell wall chitin, followed by the reactivation of β-1,3-glucan synthase (Wagener and Loiko, 2018). In this study, although the highest dose reduced efficacy, mycelial growth remained minimal with noticeable morphological differences compared to the control. Degradation of biocontrol products may also play a role. For example, *Beauveria bassiana* can tolerate, bioaccumulate, and solubilize copper through mechanisms such as cell wall binding, cellular absorption, chelation, and precipitation *via* secreted molecules (Cervantes and Gutierrez-Corona, 1994; Martins et al., 2012). To eliminate the possibility of potential adaptation, successive transplanting experiments conducted in the presence of this biocontrol product could serve as a valuable approach (Yang et al., 2008; Ajouz et al., 2010; Childers et al., 2015), even though the adaptation to a plant extract-based biocontrol product with a multiple mode of action is not well recognized in the literature (Bardin et al., 2015). The reduced efficacy observed at the highest FV doses suggests that further investigations are needed to assess whether prolonged exposure could lead to tolerance or resistance mechanisms in *P. infestans.* Future investigations should focus on mycelial viability, potential shifts in sensitivity over successive generations, and product quantification in both the mycelium and the culture medium. However, the complexity of the formulation may present challenges.

### 4.2 Impact of FV and PE on *Phytophthora infestans* infection structures

The two infection structures of *P. infestans*, sporangia and zoospores, contribute to its high infectivity and make it particularly challenging to control. Airborne sporangia can either germinate directly to penetrate the leaf cuticle or release multiple zoospores that germinate and infect the leaves (Fry, 2008; Skelsey et al., 2009). These diverse infection mechanisms highlight the necessity of targeting both structures to ensure effective oomycete control. This study demonstrated that both FV and PE have a strong impact on these infection structures. Consistent with the results from mycelial growth experiments, FV significantly affected sporangia at the lowest concentration tested. Interestingly, while PE had no impact on mycelial growth, it disturbed sporangia germination at a concentration of 0.10%. An important difference between the two tests is the nature of contact between the sporangia and the mycelium with PE. Sporangia suspended in water are in direct contact with PE, while the mycelium grows on a nutrient medium containing PE. In the latter case, the contact might be reduced, where the medium might represent an obstacle for direct contact between the mycelium and PE. The differences or similarities in sensitivity between mycelial growth and sporangia observed in our study align with findings from several other studies. For instance, La Torre et al. (2019) demonstrated similar sensitivity between these two structures when exposed to beet (*Beta vulgaris* subsp. *Vulgaris*) molasses vinasse biocontrol products at a concentration of 0.60%. Dorn et al. (2007) demonstrated variable efficacy of certain plant extract treatments on mycelial growth and sporangia. For example, biocontrol product Inulex, based of extract of *Inula viscosa* at 1.00%, strongly inhibited mycelial growth but weakly affected sporangia germination, while *Rheum rhabarbarum* extract at 5.00% affected sporangial germination thought had no impact on mycelial growth. In this study, both sporangial germination and zoospore release were reduced at 0.01% FV and 0.10% PE, suggesting similar sensitivities of these structures to the treatments. These results had a comparable impact on these processes like other biocontrol agents, such as fengycin B, while the volatile compound 1-undecene, produced by *Pseudomonas* strains, inhibited sporangial germination at lower concentrations than those required to affect zoospore release (Hunziker et al., 2015; Wang et al., 2020a). These findings suggest that the inhibition of zoospore release and sporangial germination is dependent on the specific active substance used.

Numerous studies have demonstrated the effects of plant extract-based biocontrol products on mycelium and sporangia, but their impact on zoospores has been less explored (La Torre et al., 2019; Najdabbasi et al., 2020). Zoospores, like sporangia, are crucial for successful infection (Fry, 2008). Our results showed that while PE remained effective from 0.10%, FV inhibited zoospore germination at 0.05%, a concentration higher than that needed to affect sporangial germination. Interestingly, the germination rate of sporangia under control conditions averaged 45%, while that of zoospores was 86%. These observations are in line with previous studies, which also reported a significantly higher natural germination rate for zoospores compared to sporangia under *in vitro* conditions (Bruisson et al., 2019; De Vrieze et al., 2020). This higher germination capacity of zoospores may explain their differential susceptibility to FV. A recent study by Wu et al. (2021) has demonstrated the inhibitory effects of a *Myxococcus xanthus* extract on the overall germination of *P. infestans* zoospores. Similarly, Matheron and Porchas (2000) reported the impact of various fungicides on the zoospore’s germination from multiple *Phytophthora* species. Beyond germination rates, the phenotypes of germinating zoospores can provide critical insights (De Vrieze et al., 2020). In our study, non-inhibitory concentrations of FV and PE induced phenotypic changes with a higher occurrence of phenotypes, such as zoospores with multiple germ tubes and appressorium-like formation. Previous studies on cellulose synthase (CesA) and transglutaminase (TGase) genes, both involved in maintaining *P. infestans* cell wall integrity, also reported similar germination phenotypes, referred to as “appressorium and germ tube aberrant” (Grenville-Briggs et al., 2008; Brus-Szkalej et al., 2021). Consistent with our results, a higher proportion of these phenotypes was observed in zoospores exposed to a TGase inhibitor and in TGase or CesA silenced lines. Additionally, zoospore lines with silenced TGase or CesA genes showed reduced or no pathogenicity when inoculated on leaves compared to control lines. De Vrieze et al. (2020) also reported shifts in zoospore phenotypes when exposed to biocontrol strains of *Pseudomonas*, although the specific germination phenotypes described here were not reported. Consistent with our findings, they observed a reduction in the proportion of zoospores named regular appressorium formation when exposed to a strain of *Pseudomonas*, corresponding to the category we defined as short germ tube with appressorium formation. When exposed to inhibitory concentrations of FV and PE, the few zoospores that germinated exhibited short germ tubes and no appressoria formation. Similarly, Bruisson et al. (2019) observed abnormal germination in zoospores and reported a significant proportion of zoospores with retarded germination when incubated with some phyllosphere bacteria. These findings suggest that the biocontrol product may impair cell wall integrity, though further research is required to confirm this hypothesis.

To understand the impact of biocontrol products (FV and PE) on the infection structures, membrane integrity was monitored using propidium iodide staining (Zhu et al., 2023). Both FV and PE were found to compromise the membrane integrity of sporangia and zoospores, with zoospores exhibiting a greater sensitivity, particularly following PE treatment in comparison to sporangia. Similar findings were reported by Joller et al. (2020), though to a lesser extent. The lack of a cell wall before zoospore encystment could facilitate easier diffusion of the biocontrol product, enabling it to more effectively reach and disrupt the plasma membrane. The observed membrane integrity disruption in both infection structures at lower doses for FV supports the hypothesis that the formulation enhances the plant extract’s activity on membrane integrity. The precise mechanism underlying membrane integrity disruption has not yet been fully elucidated, although several hypotheses can be proposed based on previous studies. To test this, it would be useful to assess mycelium membrane permeability using propidium iodide (PI). Additionally, studies on the effects of amphiphilic molecules, whether of microbial or plant origin, have provided insights into these mechanisms. For instance, Botcazon et al. (2022) used Alexa Fluor 488-Annexin V staining, on *Sclerotinia sclerotiorum* mycelium, in addition to PI, which specifically binds to phosphatidylserine (PS) exposed on the outer membrane, an early marker of cell death. Recently, Wang et al. (2020b) also supported their observations on *P. infestans* mycelium by measuring electrolyte leakage into the culture medium, which can be quantified through conductivity measurements. Furthermore, additional analyses such as quantifying reactive oxygen species (ROS), measuring lipid peroxidation, and using transmission electron microscopy (TEM), would help confirm the impact of these molecules on membrane integrity (Ito et al., 2007; Wang et al., 2020b; Adamczyk et al., 2023).

### 4.3 Differential effects of FV and PE on non-target fungal and bacterial species

In this study, the non-target effects of FV and PE were assessed on three phyllosphere fungi (*Mucor* sp., *Cladosporium* sp., and *Fusarium* sp.) *and* the plant pathogen *S. sclerotiorum*. The results revealed that while all tested fungi exhibited sensitivity to both versions, this sensitivity occurred mainly at higher concentrations, with FV consistently demonstrating greater impact than PE. Among the tested fungi, *Mucor* sp. demonstrated the highest sensitivity to both versions, followed by *Cladosporium* sp., while *Fusarium* sp. and *S. sclerotiorum* were the least affected. This variation in sensitivity may reflect biochemical differences across fungal phyla (Ghormade et al., 2017; Wang et al., 2021). Our previous results on the infection structures of *P. infestans* suggest that the fungal cell wall and plasma membrane are potential primary targets of FV and PE. This may explain the heightened susceptibility of *Mucor* sp., belonging to the Mucoromycetes phylum, which has a unique cell wall composition (Dzurendova et al., 2022; Sokołowska et al., 2023). Unlike the cell walls of Ascomycetes and Basidiomycetes phylum, which predominantly contain chitin and glucans (Bowman and Free, 2006), *Mucoromycetes* cell walls are mainly composed of chitin and chitosan with minimal amounts of glucans (Bartnicki-Garcia and Reyes, 1964; Ghormade et al., 2017). Furthermore, the monosaccharide profile of Mucoromycetes cell walls, which includes fucose and glucuronic acid, contrasts with the glucose-, mannose-, and galactose-rich walls of ascomycetes and Basidiomycetes (Yugueros et al., 2024). Further investigations could clarify *Mucor* sp. susceptibility to FV and PE. Enzymatic assays (e.g., chitinases, chitosanases, and glucanases) could reveal whether the biocontrol product mimics these enzymes. Fluorescence or electron microscopy with specific stains (Calcofluor White for chitin, Aniline Blue for *β*-glucans) could identify structural alterations, while propidium iodide (PI) staining could assess membrane integrity. At the molecular level, gene expression analysis of cell wall biosynthesis and remodeling enzymes could help determine whether *Mucor* sp. sensitivity stems from intrinsic cell wall composition or differential stress adaptation mechanisms.

Differences in plasma membrane composition may also influence the response of fungi to the biocontrol product. For instance, Avis and Bélanger (2001) previously showed that fungal susceptibility to membranotropic compounds was associated with the total membrane lipid content. Among these lipids, ergosterol is a major sterol in fungal plasma membranes which is crucial for maintaining membrane integrity (Rattray et al., 1975; Avis, 2007). However, ergosterol content may vary across fungal species and isolates (Avis and Bélanger, 2001; Botcazon et al., 2022). This variation has been shown to influence fungal sensitivity to membranotropic compounds, as observed in *S. sclerotiorum* isolates (Wise et al., 2014; Botcazon et al., 2022). In our study *S. sclerotiorum* and *Fusarium* sp. were the fungi least sensitive to FV and PE, often overcoming initial inhibition at certain doses. Reduced sensitivity of *Fusarium* sp. compared to other fungi has been similarly observed in a study involving exposure to terpenes (Adamczyk et al., 2023). This resilience may be linked to their respective lifestyles: *S. sclerotiorum* is a necrotroph, while *Fusarium* spp., mostly phytopathogenic exhibits a hemibiotrophic lifestyle (Bolton et al., 2006; Pradhan et al., 2020; Nikitin et al., 2023). Fungi with these lifestyles are known to detoxify antifungal compounds via enzymatic degradation and efflux mechanisms (Westrick et al., 2021). For example, *Fusarium oxysporum* f. sp. *lycopersici* degrades the membrane-disrupting saponin *α*-tomatine into less-toxic tomatidine (Osbourn, 1996; Sandrock and VanEtten, 1997). Additionally, efflux transport proteins, such as ABC and MFS transporters, are also implicated in detoxification. In contrast, bacterial species showed differential sensitivity to FV. The Gram-positive bacterium *B. subtilis* was more susceptible than the Gram-negative *P. fluorescens*, likely due to structural differences in their cell envelopes (Beveridge, 1999). The presence of an outer lipid membrane in Gram-negative bacteria, which shields the peptidoglycan layer, likely reduces FV accessibility compared to Gram-positive bacteria, which lack this barrier (Nikaido and Nakae, 1980). While plant extracts have shown antibacterial potential (Nourbakhsh et al., 2022), some studies reported their ineffectiveness on bacterial growth (Erdogrul, 2002). Similarly, our results showed that PE had no effect on bacterial growth. The effect of FV at higher concentrations, particularly at 0.10%, on the fungus *Mucor* sp. and the bacterium *Bacillus subtilis* raises questions about their respective roles in ecosystems. The *Mucor* genus, which is predominantly saprophytic, plays a key role in organic matter decomposition and biogeochemical cycles (Hoffmann et al., 2013). *Bacillus subtilis*, in addition to being a plant growth-promoting rhizobacterium (PGPR), also exhibits biocontrol activity (Blake et al., 2021). Given the observed effects on these microorganisms, it is possible that other, more sensitive species could also be impacted. The inhibition observed in our study results from direct interaction between these microorganisms and the biocontrol product, but its broader impact on microbial dynamics within the ecosystem requires further investigation. Therefore, it is essential to conduct additional experiments to assess the effect of FV on *in planta* microbial communities, particularly through metabarcoding analysis. The phyllosphere hosts essential microbial communities, including many beneficial microorganism and natural biocontrol agents (Legein et al., 2020; Sohrabi et al., 2023). Since biocontrol products are often applied repeatedly during the crop cycle, evaluating the long-term effects of FV on the foliar microbiome is crucial. Some studies have highlighted the resilience of vineyard phyllosphere microbial communities to fungicides (Perazzolli et al., 2014). However, recent research suggests that repeated fungicide use can significantly alter the composition of soil and phyllosphere microbial communities. It is therefore essential to determine whether prolonged application of FV could induce similar changes, potentially affecting the stability and functionality of the foliar microbiome.

Comparing the impact of the biocontrol products on the mycelial growth of oomycetes, fungi, and bacteria growth, *P. infestans* demonstrated the highest sensitivity to FV compared to fungi showed the highest sensitivity to PE. However, the results obtained with the concentrated plant extract (CPE) on *P. infestans* mycelial growth presented a dose-dependent response on plant extract. Based on these findings the high efficacy of FV at low concentrations suggests the plant extract activity optimization by the formulation. Moreover, this differential sensitivity of *P. infestans* to FV and PE in comparison to the other microorganisms tested could be explained by its auxotrophic characteristic for sterols. In addition, the unique composition of *P. infestans* cell wall, which contains cellulose and lacks chitin may have also a role in this variation (Bartnicki-Garcia, 1968; Wang et al., 2021). The hypothesis considering the cell wall and plasma membrane as potential targets of biocontrol products is supported by the observed impact on infection structures. However, it is possible that the biocontrol products also interact with other cellular components. Numerous studies have reported non-target effects of plant extracts and plant-derived compounds, meanwhile our findings suggest that the biocontrol product has minimal non-target effects, particularly at low doses (Adamczyk et al., 2023; Teixeira et al., 2023). The plant extract showed efficacy against *P. infestans* infection structures at a dose (0.10%) where bacterial and fungal growth was not compromised. The formulated version provided better efficacy against *P. infestans*, but a moderate impact was observed from 0.10% on bacterial growth of *B. subtilis* and *Mucor* sp. This study shows that the choice of dose is crucial to control *P. infestans* infection while avoiding a non-target effect on microorganisms in the phyllosphere. To achieve a better balance between efficacy and safety, work on formulation might be also a determining factor to limit at the maximum the impact on non-target microorganisms.

In conclusion, this study underscores *in vitro* efficacy of the low-dose biocontrol product against *P. infestans*, including its infection structures and mycelial growth, while demonstrating minimal impact on non-target microorganisms. The addition of co-formulants appears to be a promising approach for enhancing the activity of existing plant extracts, highlighting the need for further investigation to better understand the underlying mechanisms. Future research should aim to elucidate the mode of action of the plant extract and its formulated version, assess their effects on microbial diversity in plant and soil ecosystems, and explore their potential to induce plant resistance against late blight.

## Data Availability

The raw data supporting the conclusions of this article will be made available by the authors, without undue reservation.
