# Supplementary material for: In vitro biocontrol potential of plant extract-based formulation against infection structures of Phytophthora infestans along with lower non-target effects
Source: Front Microbiol. 2025 Apr 14;16:1569281. doi: 10.3389/fmicb.2025.1569281 (PMC12034721; doi:10.3389/fmicb.2025.1569281)
Supplement: Supplementary file 1 [file Data_Sheet_1.docx]

Supplementary Material

# Supplementary Figures and Tables

## Supplementary Figures

**Supplementary Figure 1.**


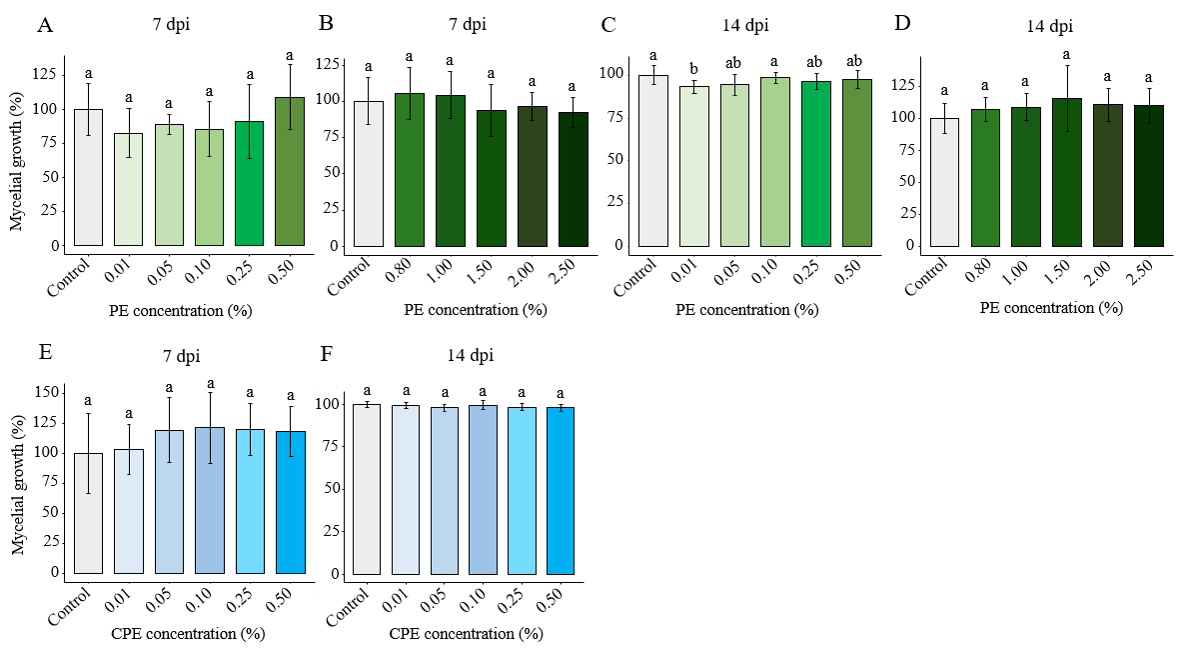
Effect of plant extract (PE) and concentrated plant extract (CPE) at different concentrations on *Phytophthora infestans* mycelial growth at early time (7 days post inoculation - dpi) and late time (14 dpi) (A, B, E and C, D, F respectively). The percentage of growth was calculated by setting mycelial growth in control at 100%. The bars represent the average percentage of growth with standard errors of the 10 replicates pooled (from 2 independent experiments, each with 5 replicates). Letters indicate significant differences between treatments (Kruskal-Wallis test, and Wilcoxon rank-sum test for multiple comparisons, *P* ≤ 0.05, *n* = 10).

**Supplementary Figure 2.**

Representative pictures of sporangia in the presence of different doses of plant extract (PE), concentrated plant extract (CPE), and formulated version (FV). Pictures were captured in bright field (BF) and red fluorescent protein (RFP) channel at 10x magnification using an inverted epifluorescence microscope (EVOS M5000, Invitrogen Thermo Fisher Scientific). Scale bars indicate 100 µm.


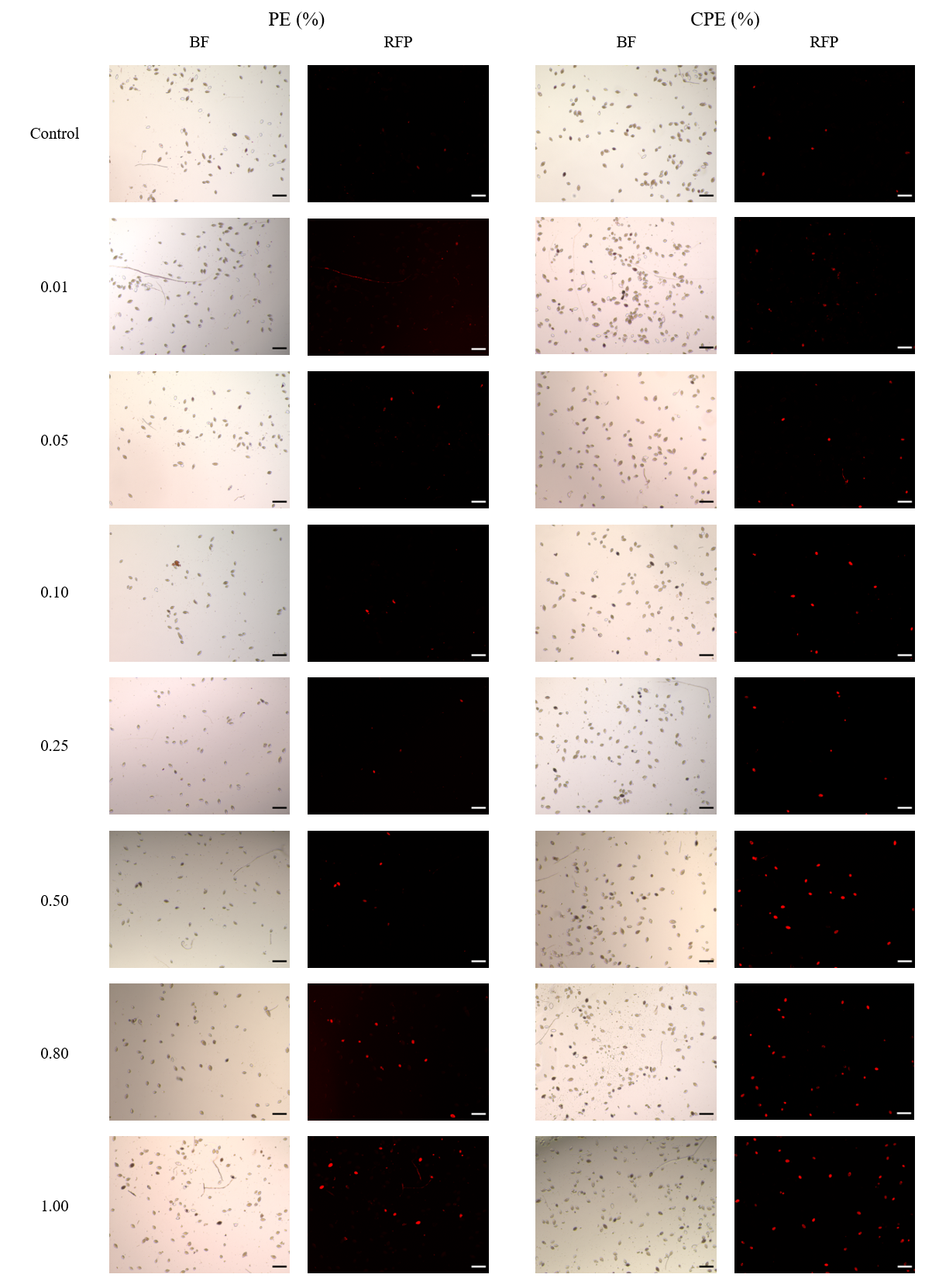


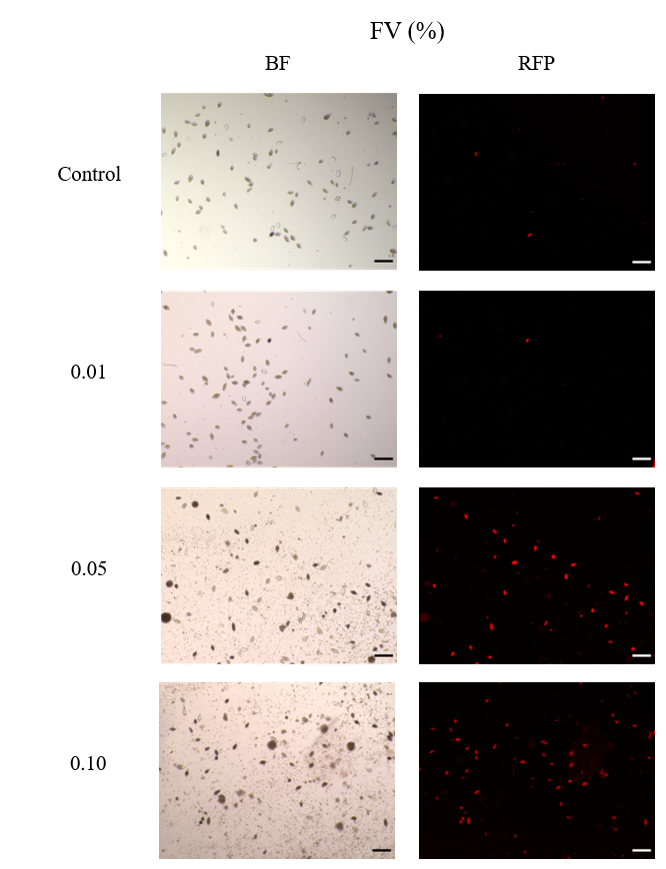


**Supplementary Figure 3.**

Representative pictures by time and different dose of plant extract (PE) and formulated version (FV). The early time corresponds to 2-7 days post-inoculation (dpi) and late time to 4-14 dpi. These time points depend on the mycelial growth rate of the two fungi (refer to the Materials and methods section).


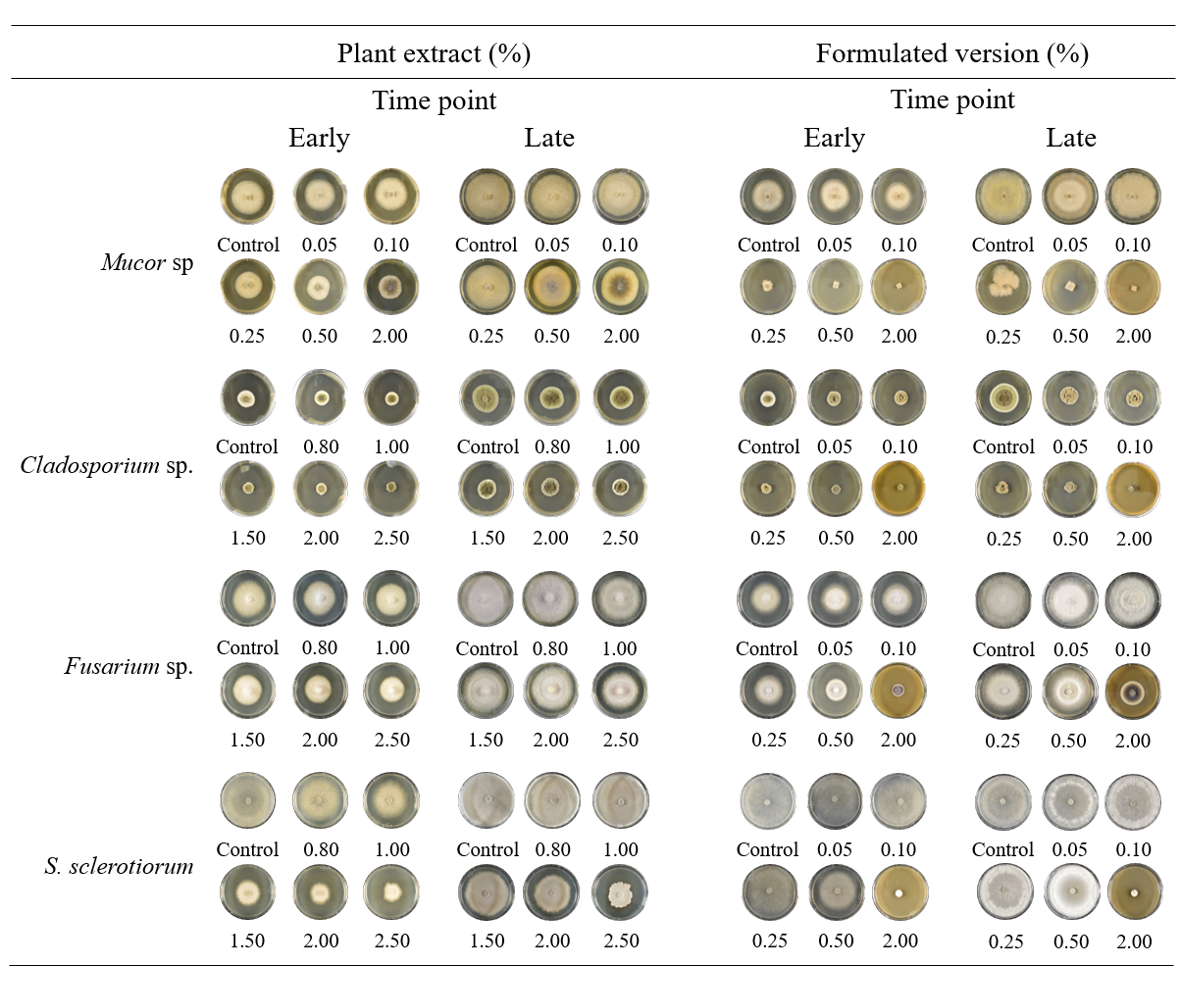


**Supplementary Table S1**

Effect of plant extract (PE) at different concentrations on the mycelial growth of *Cladosporium* sp., *Fusarium* sp. and *S. sclerotiorum* at early time and late time. The early time corresponds to 2-7 days post-inoculation (dpi) and the later time to 4-14 dpi. These time points depend on the mycelial growth rate of the fungi (refer to Materials and methods section). The percentage of growth was calculated by setting mycelial growth in controls at 100%. Values represent the average percentage growth with standard errors of the 10 replicates pooled (from 2 independent experiments, each with 5 replicates). The color scale indicates the percentage of mycelial growth (dark red: high inhibition; white: no inhibition). Letters indicate significant differences between treatments (Kruskal-Wallis test, and Wilcoxon rank-sum test for multiple comparisons test, *P* ≤ 0.05, *n* = 10)

|  | Plant extract (%) | | | | |
| --- | --- | --- | --- | --- | --- |
|  |  |  |  |  |  |
|  | Timepoint | | | | |
|  |  | Early |  | Late |  |
| *Cladosporium* sp. | Control | 100.0 ± 6 | a | 100.0 ± 13 | a |
|  | 0.05 | 106.2 ± 22 | a | 133.1 ± 63 | a |
|  | 0.10 | 94.1 ± 10 | ab | 122.3 ± 40 | a |
|  | 0.25 | 90.6 ± 14 | ab | 124.1 ± 26 | a |
|  | 0.50 | 79.9 ± 9 | b | 97.6 ± 17 | a |
|  |  |  |  |  |  |
| *Fusarium* sp*.* | Control | 100.0 ± 5 | a | 100.0 ± 6 | a |
|  | 0.05 | 88.9 ± 5 | a | 101.1 ± 10 | a |
|  | 0.10 | 93.3 ± 5 | a | 103.4 ± 5 | a |
|  | 0.25 | 92.2 ± 7 | a | 103.9 ± 5 | a |
|  | 0.50 | 93.6 ± 6 | a | 97.8 ± 10 | a |
|  |  |  |  |  |  |
| *S. sclerotiorum* | Control | 100.0 ± 1 | a | 100.0 ± 1 | a |
|  | 0.05 | 100.3 ± 1 | a | 99.8 ± 1 | a |
|  | 0.10 | 99.8 ± 1 | a | 99.8 ± 1 | a |
|  | 0.25 | 99.8 ± 1 | a | 99.9 ± 1 | a |
|  | 0.50 | 100.0 ± 1 | a | 99.9 ± 1 | a |

| Mycelial growth (%) | ≥0; <20 | ≥20; <40 | ≥40; <60 | ≥60; <80 | ≥80; ≤150 |
| --- | --- | --- | --- | --- | --- |
